# Development and application of an indirect ELISA and nested PCR for the epidemiological analysis of *Klebsiella pneumoniae* among pigs in China

**DOI:** 10.3389/fmicb.2023.1329609

**Published:** 2024-01-08

**Authors:** Zengshuai Wu, Na Li, Ziheng Li, Jianlong Wang, Mengmeng Liu, Mengzhu Qi, Shaopeng Wei, Tong Wu, Yu Guo, Junhui Zhu, Hexiang Jiang, Ruixue Xue, Changjiang Sun, Xin Feng, Jingmin Gu, Wenyu Han, Fengyang Li, Liancheng Lei

**Affiliations:** ^1^State Key Laboratory for Zoonotic Diseases, College of Veterinary Medicine, Jilin University, Changchun, China; ^2^Animal Disease Control Center of Inner Mongolia, Hohhot, China; ^3^Department of First Hospital, Jilin University, Changchun, China; ^4^College of Veterinary Medicine, Shandong Agricultural University, Taian, China; ^5^Animal Disease Control Center of Shandong, Jinan, China; ^6^College of Animal Science, Yangtze University, Jingzhou, China

**Keywords:** Klebsiella pneumoniae, ELISA, nested PCR, khe, pig serum

## Abstract

**Introduction:**

*Klebsiella pneumoniae* (*K. pneumoniae*) is an important opportunistic and zoonotic pathogen which is associated with many diseases in humans and animals. However, the pathogenicity of *K. pneumoniae* has been neglected and the prevalence of *K. pneumoniae* is poorly studied due to the lack of rapid and sensitive diagnosis techniques.

**Methods:**

In this study, we infected mice and pigs with *K. pneumoniae* strain from a human patient. An indirect ELISA was established using the KHE protein as the coating protein for the detection of *K. pneumoniae* specific antibody in clinical samples. A nested PCR method to detect nuclei acids of *K. pneumoniae* was also developed.

**Results:**

We showed that infection with *K. pneumoniae* strain from a human patient led to mild lung injury of pigs. For the ELISA, the optimal coating concentration of KHE protein was 10 µg/mL. The optimal dilutions of serum samples and secondary antibody were 1:100 and 1:2500, respectively. The analytical sensitivity was 1:800, with no cross-reaction between the coated antigen and porcine serum positive for antibodies against other bacteria. The intra-assay and inter-assay reproducibility coefficients of variation are less than 10%. Detection of 920 clinical porcine serum samples revealed a high *K. pneumoniae* infection rate by established indirect ELISA (27.28%) and nested PCR (19.13%). Moreover, correlation analysis demonstrated infection rate is positively correlated with gross population, Gross Domestic Product (GDP), and domestic tourists.

**Discussion:**

In conclusion, *K. pneumoniae* is highly prevalent among pigs in China. Our study highlights the role of *K. pneumoniae* in pig health, which provides a reference for the prevention and control of diseases associated with *K. pneumoniae*.

## 1 Introduction

*Klebsiella pneumoniae* (*K. pneumoniae*) is a rod-shaped gram-negative and opportunistic bacterium belongs to the *Enterobacteriaceae* family. This organism inhabits diverse environments including soil, water, plants, animals and humans (Yang Y. et al., 2019; Ribeiro et al., 2022). As an important opportunistic pathogen, it resides in the respiratory tract and intestinal tract of humans and animals and causes serious infections including pneumonia, septicemia, urinary tract infection, and traumatic infection (Paczosa and Mecsas, 2016).

In recent years, hypervirulent *K. pneumoniae* (hvKP) and multi-drug resistant *K. pneumoniae*, such as carbapenem-resistant *K. pneumoniae* (CRKP), have emerged and led to serious life-threatening community-and hospital-acquired infections worldwide, which makes it a global public health threat (Wang et al., 2020; Jin et al., 2022). In veterinary medicine, there are only a few reports on infections caused by *K. pneumoniae*. For example, *K. pneumoniae* causes respiratory distress in minks (Jian-Li et al., 2017), pneumonia in sheep and goats (Mahrous et al., 2023), clinical mastitis in cow (Yang Y. et al., 2019), and septicemia in pigs (Bowring et al., 2017; Bidewell et al., 2018). However, the virulence and prevalence of *K. pneumoniae* infection in pigs are rarely reported. With the isolation of hvKP and CRKP from pig farms in many countries recently (Lalruatdiki et al., 2018; Liu et al., 2019; Mobasseri et al., 2019; Leangapichart et al., 2021; Zhao et al., 2021), whether there is a *K. pneumoniae* epidemic circulation between human beings and pigs has attracted people’s attention. And because the infections with *K. pneumoniae* are usually not fatal and sporadically in most cases (Bowring et al., 2017), pigs may tolerate or only a few are severely infected, the pathogenicity and prevalence of *K. pneumoniae* in pigs have been neglected in veterinary medicine. Considering that *K. pneumoniae* may transmit between humans and animals, the domestic animals may represent a source of the pathogenic and multidrug-resistant *K. pneumoniae* to humans (Yang F. et al., 2019; Leangapichart et al., 2021). Thus, clarifying the pathogenicity and prevalence of *K. pneumoniae* in pig herds has significant public health importance.

Many methods for the detection of *K. pneumoniae* have been reported. Conventional phenotype-based methods including microscopic examination, biochemical identification, and automatic bacterial identification apparatus such as the VITEK 2 system, are time-consuming and have low sensitivity (Dong et al., 2015; Ko et al., 2017). On the other hand, genotype-based techniques including conventional PCR (Liu et al., 2008; Turton et al., 2010), triplex PCR (Jeong et al., 2013), real-time PCR (Barbier et al., 2020; Lim et al., 2021), as well as other novel methods including matrix-assisted laser desorption/ionization time of flight mass spectrometry (MALDI-TOF MS) (Huang et al., 2022) and the loop mediated isothermal amplification (LAMP) (Dong et al., 2015; Poirier et al., 2021) have also been developed for the detection of *K. pneumoniae* in hospitals and laboratories with high sensitivity and specificity. However, some of these techniques have their own drawbacks, such as the requirement of expensive instruments, trained personnel, could not be conducted on-site, or not ideally suitable for detection of *K. pneumoniae* from serum samples (Kim et al., 2022).

Enzyme-linked immunosorbent assay (ELISA) is one of the commonly utilized serological detection methods in veterinary medicine. Although this method also has certain disadvantages listed above, it is an efficient, sensitive, specific, and convenient tool for detection of antigen or antibody levels in serum samples. However, establishment of ELISA and its application to detect *K. pneumoniae* in pigs has not been reported. A gene designated *khe*, which encodes a hemolysin of *K. pneumoniae*, has been proposed as a potential species-specific gene probe (Yin-Ching et al., 2002) and was utilized to detect the signature for clinical *K. pneumoniae* isolates from hospitals (Escobar Pérez et al., 2013; Chen et al., 2014; He et al., 2016). Li et al. (2020) designed a multiplex PCR method to detect *K. pneumoniae* in minks with the *khe* gene. These studies suggested that *khe* is a specific gene of *K. pneumoniae*.

Since there are few severely clinical symptoms, infections with *K. pneumoniae* in pigs are rarely reported and the potential harmfulness to pig health are neglected. Thus, *K. pneumoniae* may be overlooked by conventional methods for pathogen isolation and identification. Moreover, there are no vaccines against *K. pneumoniae* in pigs in China. To clarify the virulence and prevalence of *K. pneumoniae* in pigs in China, infection model by a human origin *K. pneumoniae* was constructed both in mice and pigs and an indirect ELISA was successfully established using recombinant KHE protein as the primary coating antigen. We demonstrated that *K. pneumoniae* infection led to mild lung injury in mice and pigs. In combination with a nested PCR targeting *khe*, the established methods demonstrated a high infection of *K. pneumoniae* in pigs, which is positively correlated with gross population, Gross Domestic Product (GDP), and domestic tourists, indicating a potential transmission of *K. pneumoniae* from humans to pigs.

## 2 Materials and methods

### 2.1 Bacterial strains and growth conditions

Supplementary Table S1 contains a list of every strain utilized in this investigation. Mass spectrometry and PCR were used to identify the *K. pneumoniae* strains KP-Q1 (Strain number: CCTCC PB 2023065, ampicillin resistant) and K36 (Strain number: CCTCC PB 2023066) (Cai et al., 2018), which were isolated from First Hospital of Jilin University. *K. pneumoniae* KP-Q1 was cloned by cultivating it overnight at 37°C in Luria-Bertani (LB) liquid medium supplemented with antibiotics (ampicillin, 100 μg/mL). *K. pneumoniae* K36 was cultivated in LB without antibiotics.

### 2.2 Animal experiment

Six 4-week-old female KM mice (weighing 18–20 g, free from *K. pneumoniae*, purchased from Changsheng Biotechnology) were objectively randomized into two groups (*n* = 3): a healthy control group and a *K. pneumoniae* K36 infection group. In healthy control group, mice received 40 μL PBS via intranasal injection, while mice of K36 infection group received 40 μL PBS with 8 × 10^4^ CFU *K. pneumoniae* K36. Clinical symptoms including weight, appetite and mental status were observed for 72 h post infection. A portion of the lung tissue was taken for hematoxylin–eosin staining.

Eight 45-day-old healthy Rongchang pigs (12 ~ 14 kg, free from *K. pneumoniae*, purchased from Harbin Veterinary Research Institute) were randomly assigned to two groups (*n* = 4): a healthy control group and a *K. pneumoniae* K36 infection group. In healthy control group, pigs received 1 mL PBS, while K36 infection group pigs received 1 mL PBS with 2 × 10^7^ CFU *K. pneumoniae* K36, both via intranasal injection. Clinical symptoms including appetite, mental status and body weight were continuously monitored for 96 h post infection. A portion of the lung tissue was taken for hematoxylin–eosin staining.

All mice and pigs were housed in the laboratory animal room and maintained on a 14/10-h light–dark cycle with food and water *ad libitum*. All animal experiments were approved by the Institutional Animal Care and Use Committee of Jilin University and conducted in accordance with the Chinese Laboratory Animal Administration Act 1988.

### 2.3 Serum samples collection

A total of 920 serum samples, including 434 samples from 10 pig farms located in Jinan, Shandong, China, and 486 samples from 6 cities in Inner Mongolia, China, were collected. The *K. pneumoniae* antibody-positive and-negative clinical samples for the determination of cut-off value, sensitivity, and reproducibility analysis, and the positive serums of *Actinobacillus pleuropneumoniae* (APP), *Strptococcus suis* (*S. suis*), *Staphylococcus aureus* (*S. aureus*), and *Escherichia coli* (*E. coli*) for specificity analysis of the established ELISA were collected and confirmed using PCR and sequencing and were stored at −80°C in our laboratory.

### 2.4 Purification of recombinant KHE protein

For plasmid construction, *khe* gene was amplified by PCR from the *K. pneumoniae* KP-Q1 chromosome using the primer pair KHE-F and KHE-R, which were designed by SnapGene software.^1^ Subsequently, the PCR products were digested with HindIII/XhoI restriction enzymes (Takara, Beijing, China) and ligated into the pET28a expression vector using T4 DNA Ligase (Takara, Beijing, China) and transformed into *E. coli* BL21 (DE3) cells (Sangon Biotech, Shanghai, China). Inserted DNA sequences were confirmed by DNA sequencing. Primers and plasmids used in this study are listed in Supplementary Table S1.

For protein purification, cells were cultured in LB medium at 37°C to an optical density at 600 nm (OD_600_) of 0.6 ~ 0.8, treated with 0.1 mM isopropyl β-D-1-thiogalactopyranoside (IPTG) and further incubated at 16°C for 24 h. The cells were lysed in lysis buffer (50 mM Na_2_HPO_4_ pH 7.5, 300 mM NaCl, 1 mM phenylmethylsulfonyl fluoride) by sonication. After centrifugation, the supernatant was passed through a nickel-nitrilotriacetic acid (Ni-NTA) column (GeneScript, Nanjing, China). Subsequently, the resin was washed with a 5-bed volume of washing buffer (50 mM imidazole, 50 mM Na_2_HPO_4_ pH 7.5, 300 mM NaCl) and the bound proteins were eluted with elution buffer (500 mM imidazole, 50 mM Na_2_HPO_4_ pH 7.5, 300 mM NaCl). The eluted protein was dialyzed against washing buffer using Amicon Ultra-15 filter units (Merck, New Jersey, USA) (10 kDa cut-off) and then stored in aliquots at −80°C. The entire purification process was performed on ice. The concentration of target protein was determined by a BCA protein quantification kit (Thermo Fisher Scientific, Waltham, USA). The purified proteins were analyzed using Coomassie Blue stained SDS-PAGE. Purified recombinant His-tagged KHE (rKHE) protein was further identified using anti-His antibody and *K. pneumoniae* antibody-positive serum as the primary antibody by Western blot.

### 2.5 Western blot analysis

Western blot was used to identify the purified rKHE. Firstly, purified rKHE was resuspended in SDS sample buffer and heated to 95°C for 10 min before being fractionated by SDS-PAGE electrophoresis (5% stacking gel and 12% resolving gel). Then, the proteins in the resolving gel were transferred onto a polyvinylidene fluoride (PVDF) membrane (Millipore, Massachusetts, USA). The membrane was blocked with 5% skim milk (Becton, New Jersey, USA) overnight. After that, it was incubated with mouse anti-His monoclonal antibody (1:2000; Proteintech, Chicago, USA) followed by incubation with horseradish peroxidase (HRP)-conjugated goat anti-mouse IgG (1:5000; Abconal, Wuhan, China) secondary antibody; or *K. pneumoniae* antibody positive serum (1:200) followed by incubation with goat anti-pig IgG/HRP (1:5000; Solarbio, Beijing, China) secondary antibody. Finally, the targeted proteins were visualized using the ECL reagent (Millipore, Massachusetts, USA) and a Chemiluminescent Imaging System (Tanon 5,200 multi, Shanghai, China).

### 2.6 Establishment and optimization of *Klebsiella pneumoniae* serum IgG-ELISA

The *K. pneumoniae* serum IgG-ELISA was optimized. To begin with, 100 μL of rKHE protein (1.25 μg/mL ~ 20 μg/mL) diluted with coating buffer (0.05 M carbonate buffer, pH 9.6) was coated on microtiter plates (Nest, Wuxi, China) at 4°C overnight. The unbounded protein was discarded and the wells were washed five times with phosphate buffered saline (PBS) containing 0.05% Tween-20 (PBST). The plates were blocked with 200 μL of 5% bovine serum albumin (BSA) in PBST and incubated at 37°C for 2 h. Then 100 μL of serum (diluted from 1:100 to 1:800) was added into the wells and incubated at 37°C for 0.5 h or 1 h after washing 5 times with PBST. Similarly, 100 μL of goat anti-pig IgG/HRP secondary antibody (Solarbio, Beijing, China) (diluted from 1:2500 to 1:10000 in PBS) was added and incubated at 37°C for 0.5 h or 1 h after the same washing procedure. Subsequently, the plates were washed for 5 times and 100 μL of 3,3′,5,5′-tetramethylbenzidine (TMB) (TIANGEN BIOTECH, Beijing, China) was added and incubated at room temperature for 10 min. Lastly, the reaction was terminated by addition of 50 μL of 2 M H_2_SO_4_. An ELISA plate reader (BioTek ELx800, Vermont, USA) was used to measure the optical density at 450 nm (OD_450_). Each experiment was performed at least twice, and all samples were assayed in triplicate.

### 2.7 Determination of cut-off value, sensitivity, repeatability, and specificity of the established ELISA

Twenty standard *K. pneumoniae* antibody-negative samples were selected for ELISA detection based on the optimized coating and reaction conditions. The mean value ($\bar{x}$) and the standard deviation (SD) of OD_450_ values of the negative samples were calculated. The cut-off value was determined using the mean value ($\bar{x}$) plus 3SD of the negative samples.

The coefficient of variation (CV) of intra-and inter-assay variabilities was used to assess the reproducibility of the developed ELISA. Briefly, 6 sera were randomly selected. Three replicates of each sample were run in one batch to evaluate intra-assay (within plate) variation and 3 plates were run as separate batches to evaluate inter-assay (between runs) variation.

To evaluate the sensitivity of the developed ELISA, *K. pneumoniae* positive serum diluted at 1:100, 1:200, 1:400, 1:800, 1:1600, 1:3200, and 1:6400 in PBS were tested using the optimized working conditions. To assess the specificity of the developed ELISA, positive serum samples from APP, *S. suis*, *S. aureus*, *K. pneumoniae*, and *E. coli* were detected. *K. pneumoniae* antibody-negative and-positive serum were served as negative and positive controls, respectively.

### 2.8 Development of nested PCR

According to the gene sequence of *khe* (GenBank: KX842080.1), two pairs of primers for nested PCR were designed using Primer 5 software (Premier Biosoft, Palo Alto, CA, USA) and synthesized commercially (Kumei Biotechnology, Changchun, China) (Supplementary Table S1). For nested PCR, the nucleic acids in serum samples were isolated according to a previous study (Mayoral et al., 2005). Briefly, the serum was diluted tenfold with 0.1 M HCl–Tris buffer (pH 8.0) and incubated at 100°C for 14 min. Then, it was centrifuged at 10,000 g for 5 min and the supernatant containing nucleic acids was collected for PCR analysis.

The total volume of the two rounds of PCR reaction was 20 μL: 10 μL Premix Taq polymerase (Takara, Beijing, China), 8.6 μL ddH_2_O, 0.3 μL forward and reverse primers each, and 0.8 μL DNA template. Thermal cycler conditions consisted of pre-denaturation at 95°C for 5 min, 30 cycles of 95°C for 30 s, 60°C for 30 s, 72°C for 30 s and a final extension was performed at 72°C for 10 min; stored at 4°C in a Thermal Cycler (Life ECO, Bioer Technology, Hangzhou, China). For the second round, the product of the first round was used as the DNA template. Thermal cycler conditions consisted of pre-denaturation at 95°C for 5 min, 30 cycles of 95°C for 30 s, 55°C for 30 s, 72°C for 12 s and a final extension was performed at 72°C for 10 min; stored at 4°C. The length of the targeting sequence is 210 bp. The PCR products were analyzed on a 2% agarose gel (Biowest, Loire Valley, France) containing 0.01% Gold View І (NOVON Scientific, Beijing, China) in TAE buffer (40 mM Tris–HCl pH 8.0, 1.18 mL acetic acid, 2 mM EDTA) and visualized using a Gel Doc XR+ Gel Documentation System (Bio-Rad, Hercules, CA, USA).

### 2.9 Statistical analysis

Experimental data were statistically analyzed by GraphPad Prism (version 9.0, San Diego, CA, USA). Data are presented as the mean ± SD from three independent replicates. Repeatability analysis of the established ELISA was assessed using CV (CV = SD/mean). CV values less than 15% for the intra-plate assay was considered as accepted repeatability level for the analysis. Spearman correlation coefficient was calculated to determine the relationship between ELISA positive rate and other factors. For all analyses, a *p*-value <0.05 was regarded as statistically significant.

### 2.10 Ethical approval

All animal experiments in this study were conducted in accordance with the Chinese Laboratory Animal Administration Act 1988. The animal experiments were approved by the Institutional Animal Care and Use Committee of Jilin University in compliance with the Jilin Laboratory Animal Welfare and Ethics guidelines.

## 3 Results

### 3.1 Clinical and pathological features upon *Klebsiella pneumoniae* infection in mice

**Figure 1:**
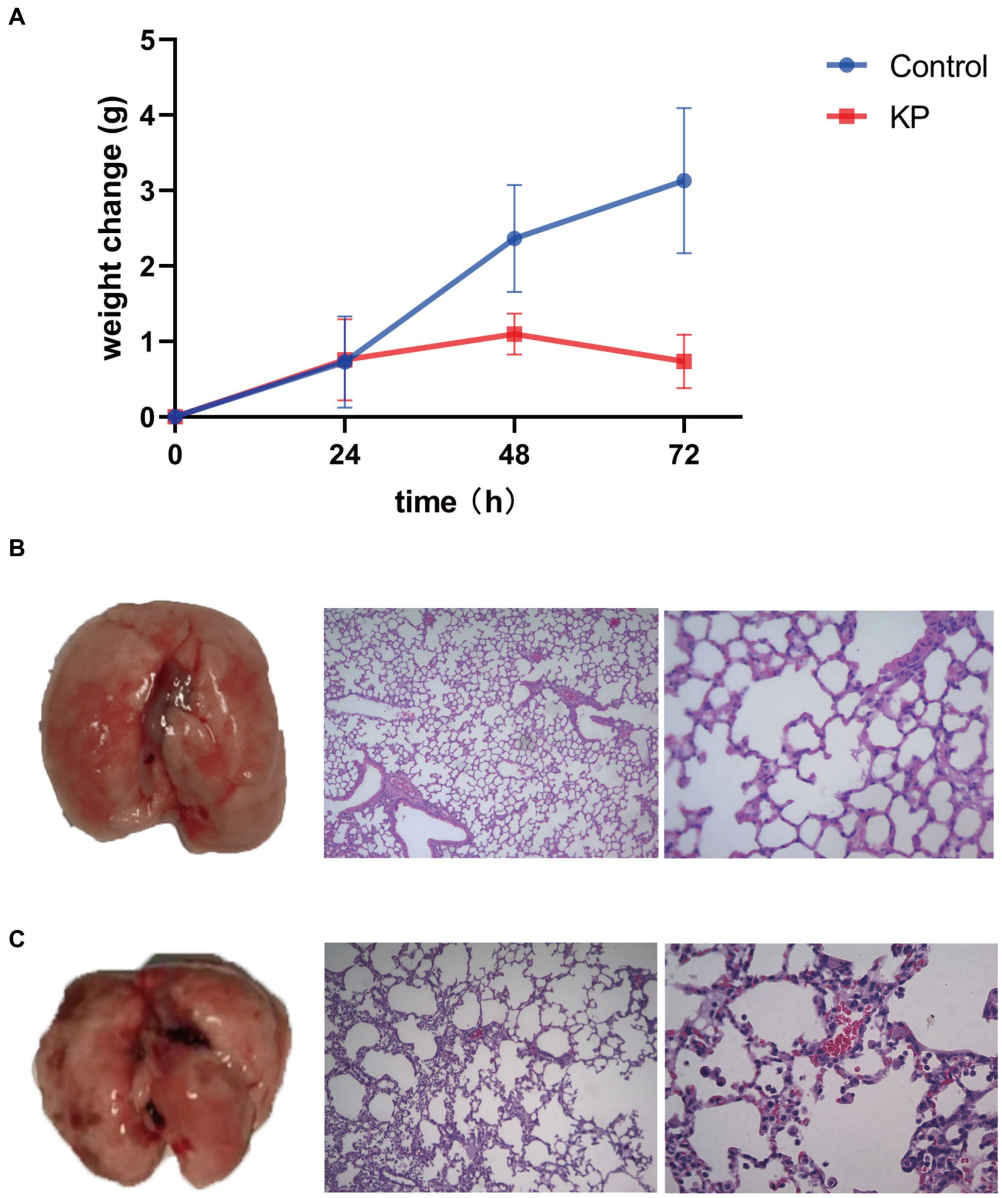
Virulence of *Klebsiella pneumoniae* in mice. **(A)** Body weight change upon infection with *K. pneumoniae* for 72 h in mice. **(B)** Left: Lung without *K. pneumoniae* infection. Middle: Hematoxylin–eosin staining of lungs of control group (100×). Right: Hematoxylin–eosin staining of lungs of control group (400×). **(C)** Left: Lung with *K. pneumoniae* infection. Middle: Hematoxylin–eosin staining of lungs of *K. pneumoniae* infection group (100×). Right: Hematoxylin–eosin staining of lungs of *K. pneumoniae* infection group (400×).

After intranasal infection with *K. pneumoniae*, the weight change of mice was significantly different at 48 h and 72 h. The weight of *K. pneumoniae* group increased slowly while the control group raised rapidly (Figure 1A). In order to further investigate the pathogenic effect of *K. pneumoniae* on mice, we euthanized the mice at 72 h. Compared with the control group of mice, the *K. pneumoniae* group exhibited significant hemorrhage, neutrophil infiltration, and alveolar septum thickening in the lungs (Figures 1B,C). These data indicate that *K. pneumoniae* can infect mice and result in lung injury.

### 3.2 Clinical and pathological features upon *Klebsiella pneumoniae* infection in pigs

**Figure 2:**
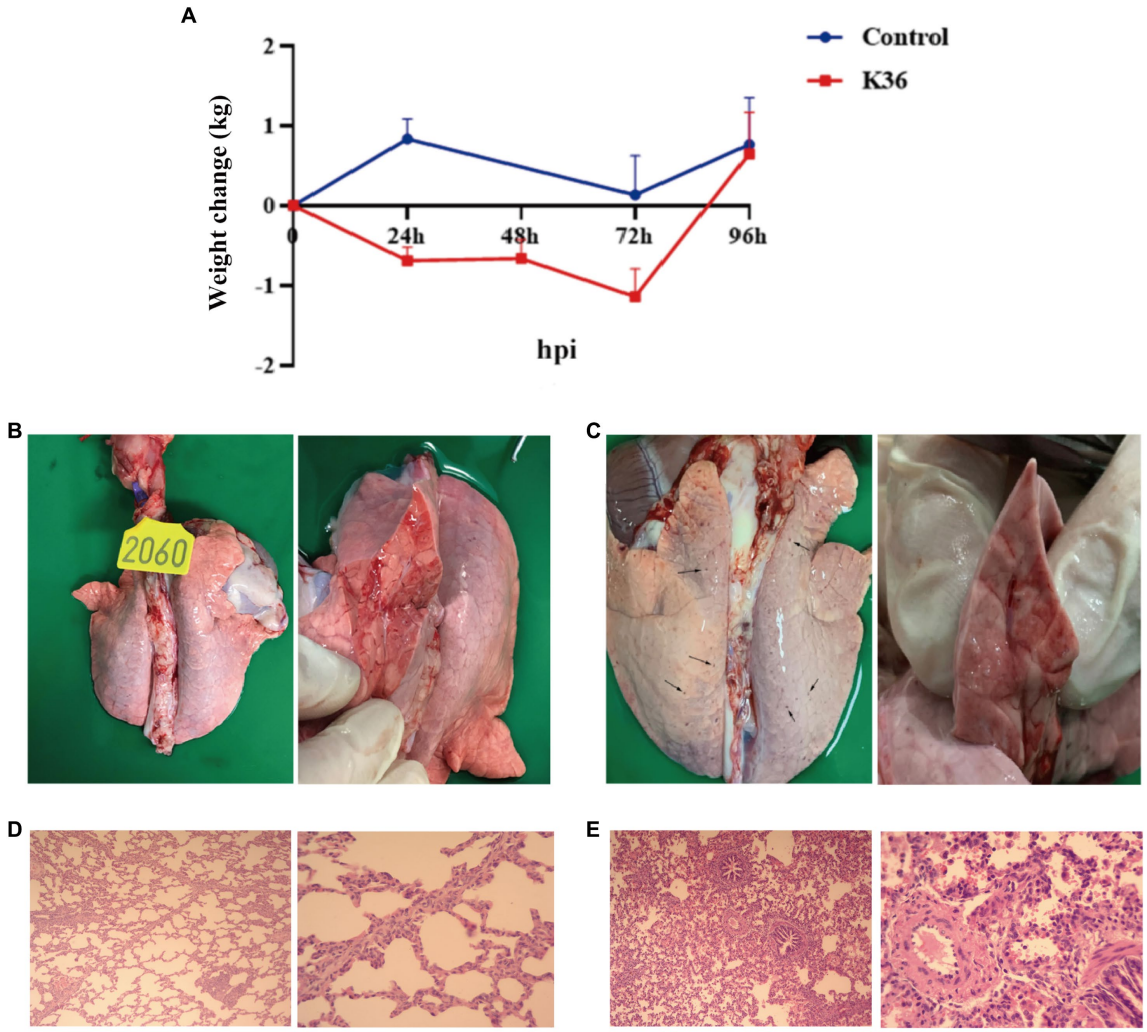
Virulence of *K. pneumoniae* in pigs. **(A)** Body weight change upon infection with *K. pneumoniae* for 96 h in Rongchang pigs. **(B,C)** Pathological examination of lung without **(B)** and with **(C)** *K. pneumoniae* infection for 96 h in Rongchang pigs. Typical petechial hemorrhage spots were indicated by black arrows. **(D)** Hematoxylin–eosin staining of lungs of control group. Left: 100×; right: 400×. **(E)** Hematoxylin–eosin staining of lungs with *K. pneumoniae* infection. Left: 100×; right: 400× .

After *K. pneumoniae* infection, only mild clinical symptoms of respiratory system, such as occasional cough and panting was were observed under our experimental condition (data not shown). However, the body weight of pigs was gradually lost during 72 h post infection, and recovered to normal at 96 h post infection (Figure 2A). To further evaluate the pathological features of *K. pneumoniae* infection, pigs were sacrificed at 96 h post infection and the lung was excised. Compared to healthy control, the lungs of *K. pneumoniae* infected pigs displayed with bilateral fleshy lesions located in the caudal lobes and cardiac notches of both lungs, without obvious boundaries between the lesions and the healthy parts. Moreover, a large number of petechial hemorrhage spots were randomly distributed on the lung surface, but not in transverse sections (Figures 2B,C). Pulmonary pathological sections indicated that *K. pneumoniae* infection group showed more obvious alveolar capillary congestion and dilation, septal thickening and inflammatory cell infiltration compared with the control group (Figures 2D,E). According to our knowledge, this is the first report on the virulence of *K. pneumoniae* on lung of pigs. These results suggest that *K. pneumoniae* infection lead to lung injury in pigs, though no obvious clinical symptoms observed.

### 3.3 Expression and purification of recombinant KHE protein

**Figure 3:**
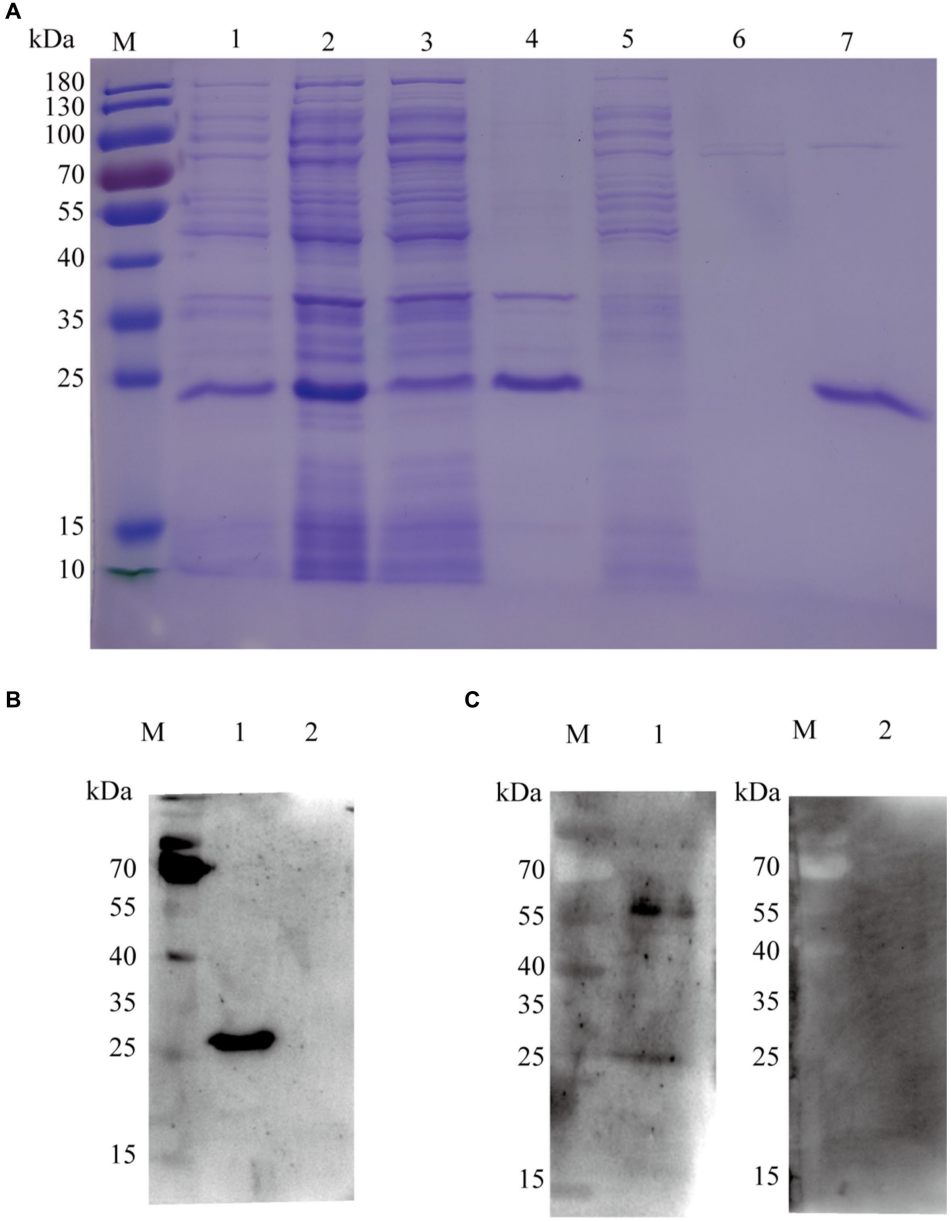
SDS-PAGE and Western blot analysis of purified rKHE. **(A)** Assessment of rKHE expression in *E. coli* by SDS-PAGE. M, Protein marker; 1, total protein without IPTG induction; 2, total protein after IPTG induction; 3, supernatant of *E. coli* lysed cells; 4, sediments of *E. coli* lysed cells; 5, 20 mM imidazole effluent; 6, 50 mM imidazole effluent; 7, purified protein; **(B)** rKHE was identified using anti-His monoclonal antibody by Western blot. M, protein marker; 1, BL21 pET28a::KHE upon IPTG induction; 2, BL21 pET28a upon IPTG induction; **(C)** Analysis of antigen reactivity of rKHE protein by Western blot. M, protein marker; 1, *K. pneumoniae*-positive serum; 2, *K. pneumoniae*-negative serum.

The expression profile of the KHE recombinant protein (rKHE, 24 kDa) was analyzed by SDS-PAGE. The results showed that it was successfully expressed and purified with high purity using a Ni-NTA resin, which was also further confirmed by Western blot analysis of purified protein using anti-His primary antibody (Figures 3A,B). Subsequently, the antigen reactivity of rKHE protein was assessed by Western blot analysis. The result showed that the rKHE protein could react specifically with the *K. pneumoniae*-positive serum (Figure 3C), indicating that the purified rKHE protein can be used as the antigen to screen specific antibodies for establishment of ELISA.

### 3.4 Establishment and optimization of the ELISA

**Figure 4:**
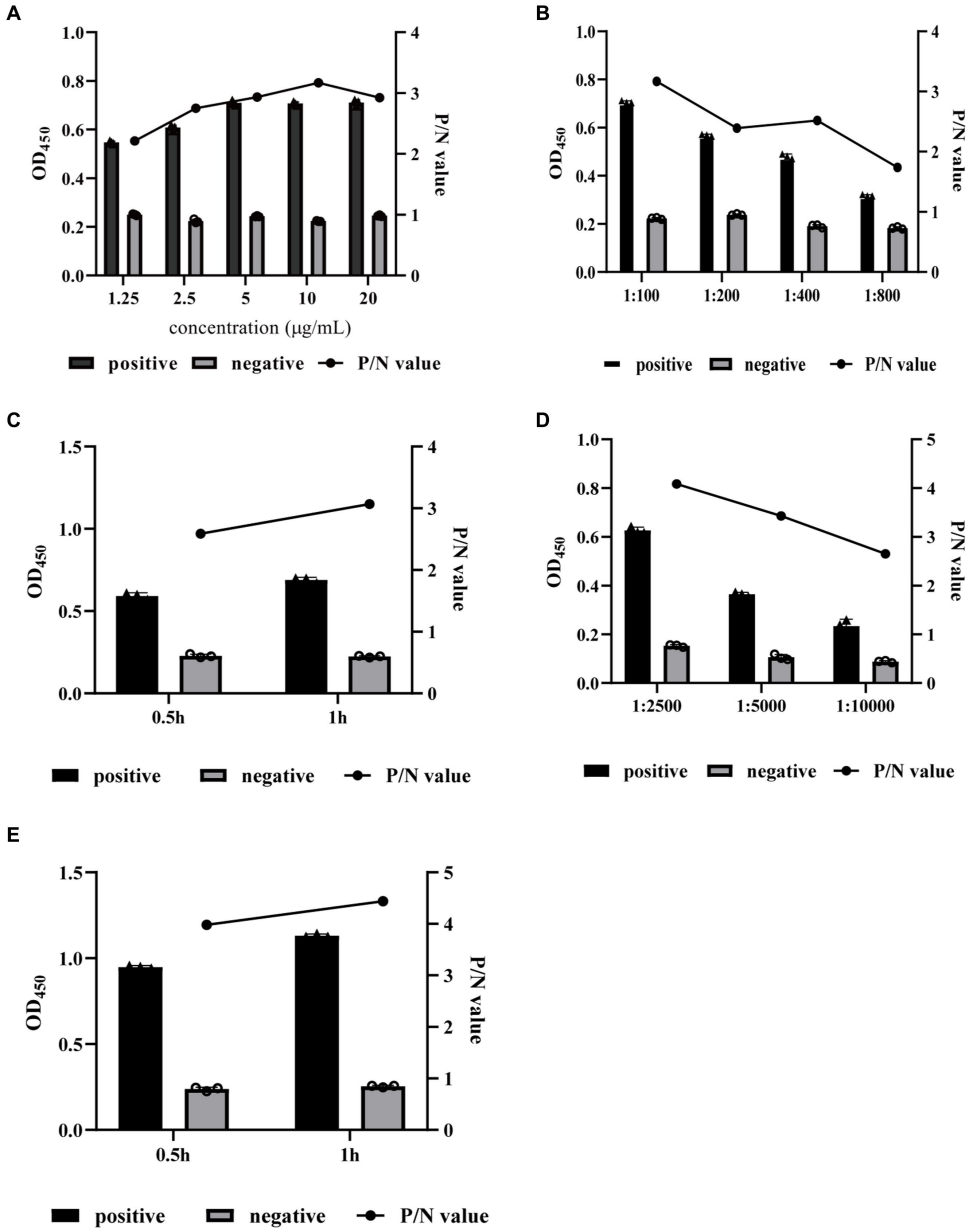
Optimization of indirect ELISA conditions. The coating concentration of rKHE antigen **(A)**, the dilution and incubation time of serum **(B,C)**, and the IgG-HRP secondary antibody **(D,E)** were optimized by checkerboard titration and selected according to the P/N value. Data are presented as the mean ± SD from three independent replicates.

Checkerboard titration was performed for the optimization of the coating concentration of rKHE antigen, the dilution and incubation time of serum and the IgG-HRP secondary antibody. The results showed that the OD_450_ value gave the maximum difference between the positive serum and negative serum (P/N value of 3.170) when the coating concentration of antigen was 10 μg/well and the dilution of serum was 1:100 (Figures 4A,B). Similarly, the optimal incubation time with the sample was 1 h (Figure 4C); the optimal dilution and the optimal incubation time of the HRP-conjugated goat anti-pig IgG secondary antibody were 1:2500 and 1 h, respectively. (Figures 4D,E).

### 3.5 Determination of cut-off value of established ELISA

The cut-off value of the established ELISA was determined by measuring the OD_450_ values of 20 standard *K. pneumoniae* antibody-negative serum samples. The results showed that the mean of percent inhibition (PI) ($\bar{x}$) of the negative serum samples was 0.231, and the SD was 0.0526. After calculation, the cut-off value of the established ELISA was 0.389. So, sample was regarded as *K. pneumoniae* antibody-positive and-negative at PI ≥0.389 and PI <0.389, respectively.

### 3.6 Determination of sensitivity, reproducibility, and specificity of established ELISA

**Figure 5:**
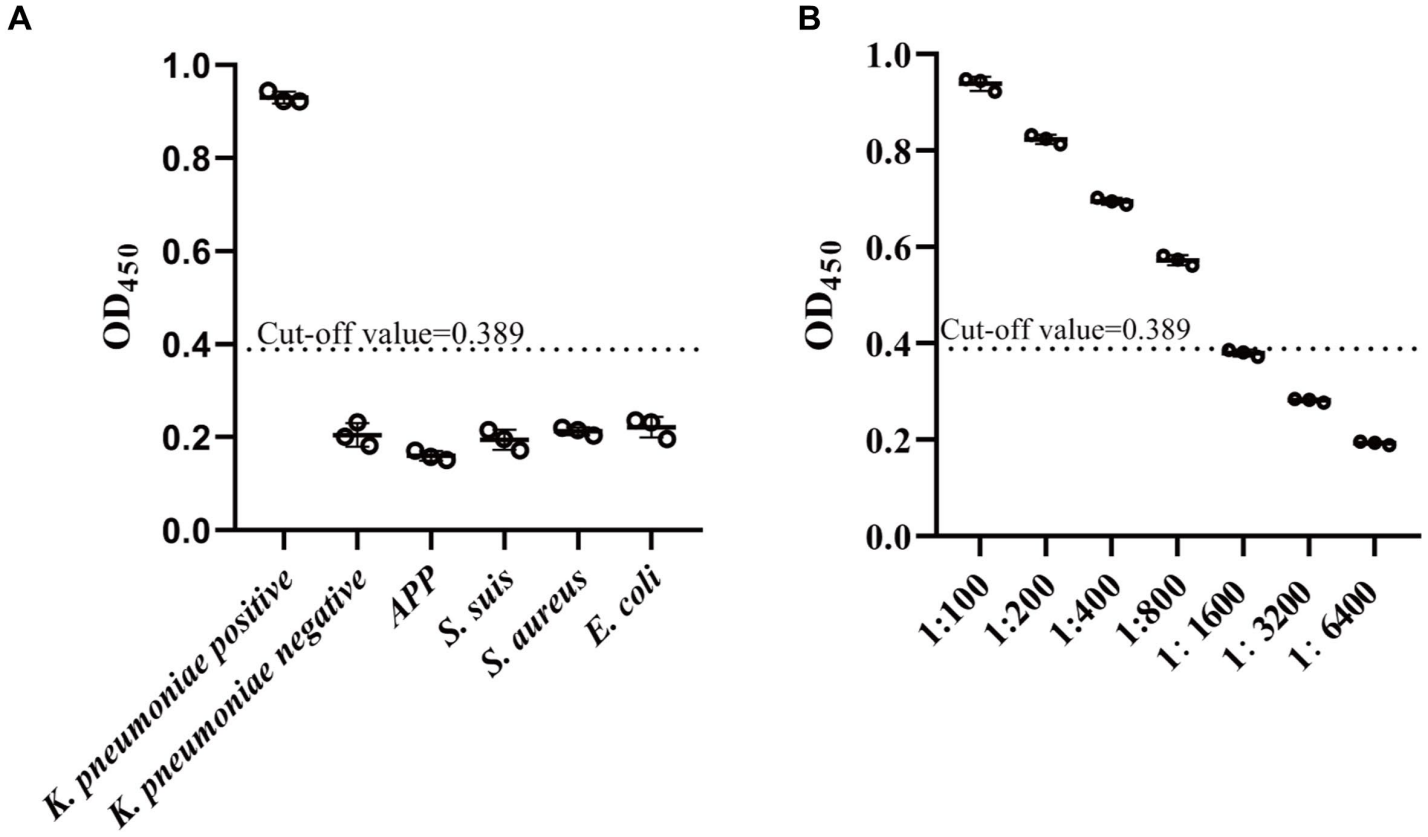
Specificity and sensitivity analysis of established indirect ELISA. **(A)** The specificity of established ELISA was evaluated by detection of cross reactions with serum containing antibodies against four common pathogens in swine, including APP (*Actinobacillus pleuropneumoniae*), *S. suis* (*Strptococcus suis*), *S. aureus* (*Staphylococcus aureus*), and *E. coli* (*Escherichia coli*). **(B)** The sensitivity of established ELISA was determined by assessment of serial dilutions of *K. pneumoniae*-positive serum. The cut-off value was determined using the mean value ($\bar{x}$) plus 3SD of the *K. pneumoniae*-negative samples. Values above cut-off was accepted as *K. pneumoniae* antibody-positive. Data are presented as the mean ± SD from three independent replicates.

The sensitivity of the established ELISA was assessed by measuring the OD_450_ values of serial dilutions of *K. pneumoniae* antibody-positive serums. The results showed that serum at a dilution of 1:800 was still *K. pneumoniae* antibody-positive, while dilution at 1:1600 was *K. pneumoniae* antibody-negative, indicating that the established indirect ELISA had a high sensitivity (Figure 5A). The CVs of intra-and inter-plate variation was used to determine the reproducibility of the established ELISA. The CVs of intra-and inter-assay ranged from 1.10 to 4.32% and 1.46 to 8.03%, respectively (Tables 1, 2), suggesting a good reproducibility. Moreover, to detect the specificity of the established ELISA, cross-reactivities with confirmed positive sera to other common swine pathogens, including APP, *S. suis*, *S. aureus*, and *E. coli*, were detected. The results showed that all gave values, except for positive control, were below the defined cut-off point (Figure 5B), indicating there’s no cross-reactivities with sera-containing antibodies against other pathogens.

**Table 1:** Intra-assay of established indirect ELISA.

Serum No.	Number of repetitions (OD_450_ values)			X^†^	SD^†^	CV%^†^
	1	2	3			
1	0.913	0.925	0.905	0.914	0.010	1.101%
2	0.179	0.171	0.175	0.175	0.004	2.286%
3	0.442	0.474	0.479	0.465	0.020	4.317%
4	0.237	0.244	0.233	0.238	0.006	2.339%
5	0.189	0.185	0.183	0.186	0.003	1.645%
6	0.165	0.161	0.173	0.166	0.006	3.673%

^†^X, average; SD, standard deviation; CV, coefficient of variation, calculated by SD divided by mean values.

**Table 2:** Inter-assay of established indirect ELISA.

Serum No.	Number of repetitions (OD_450_ values)			X^†^	SD^†^	CV%^†^
	1	2	3			
1	0.742	0.728	0.721	0.730	0.011	1.464%
2	0.169	0.147	0.148	0.155	0.012	8.032%
3	0.811	0.854	0.856	0.840	0.025	3.025%
4	0.393	0.367	0.384	0.381	0.013	3.462%
5	0.153	0.152	0.143	0.149	0.006	3.688%
6	0.425	0.443	0.446	0.438	0.009	2.055%

^†^X, average; SD, standard deviation; CV, coefficient of variation, calculated by SD divided by mean values.

### 3.7 Detection of the prevalence of *Klebsiella pneumoniae* in pigs by established ELISA

**Figure 6:**
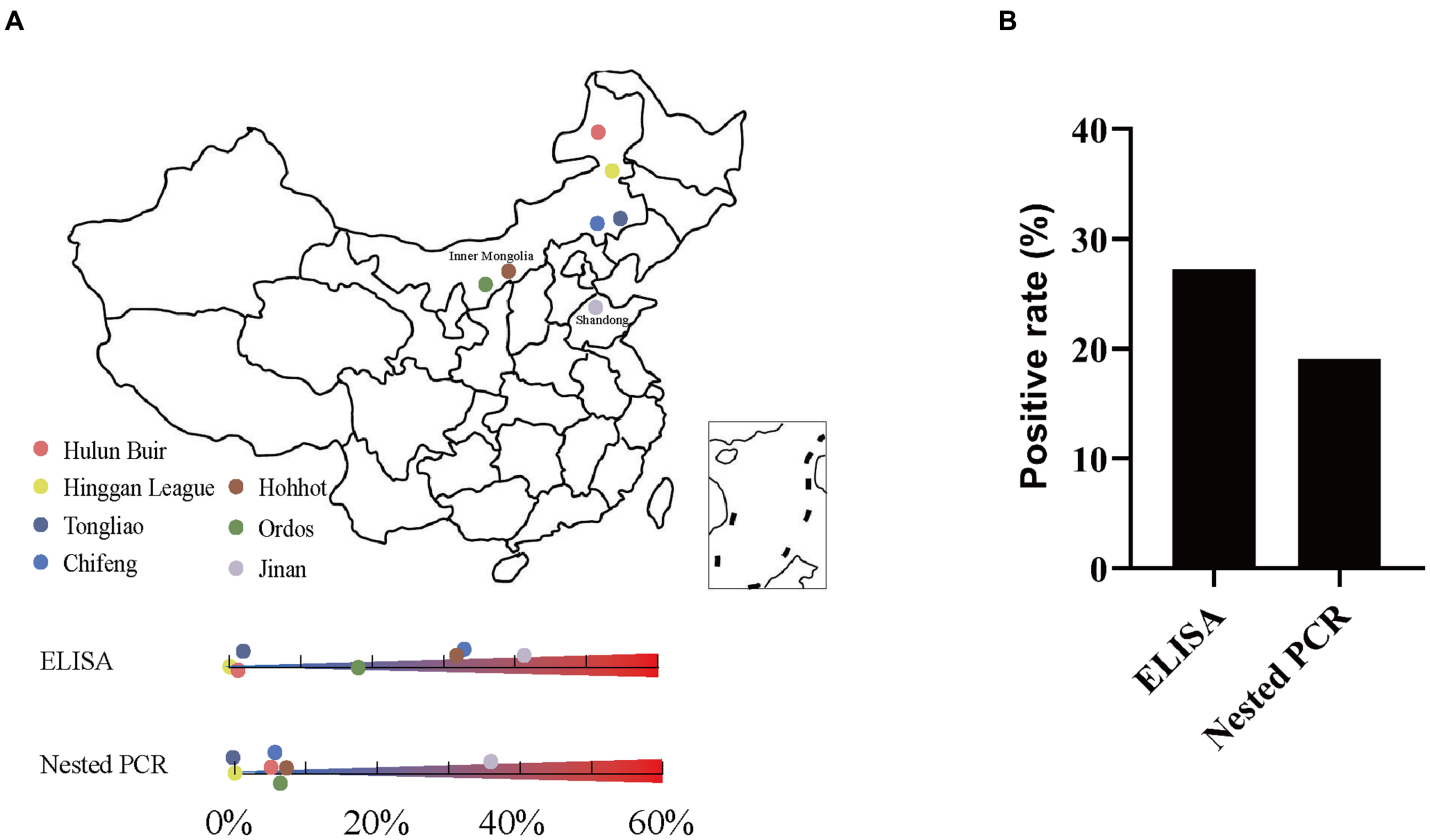
Detection of *K. pneumoniae* in serum samples by established indirect ELISA and nested PCR. **(A)** Geographic locations of pig serum samples collected from pig farms in Shandong and Inner Mongolia, China; the original map is downloaded from DataV (https://datav.aliyun.com/portal/school/atlas/area_selector). **(B)** Overall *K. pneumoniae* positive rate by established indirect ELISA and nested PCR.

A total of 920 serum samples from pig farms in Shandong and Inner Mongolia of China were investigated using the established ELISA (Figure 6A). In general, the results showed that among these samples, 251 displayed *K. pneumoniae* antibody-positive and 669 displayed *K. pneumoniae* antibody-negative, with a positive rate of 27.28% (251/920) (Figure 6B). Specifically, 179 samples from Jinan and 72 samples from Inner Mongolia showed *K. pneumoniae* antibody-positive, accounting for 41.24% (179/434) and 14.81% (72/486), respectively (Supplementary Tables S2, S3). Moreover, the positive rates of *K. pneumoniae* antibody in different cities were also different, indicating the prevalence of *K. pneumoniae* varies in different pig farms even in the same area. In pig farms of Jinan, the positive rates of *K. pneumoniae* antibody ranged from 0 to 100% (Supplementary Table S2). Interestingly, most of the pig farms from where less serum samples were collected showed a high *K. pneumoniae* antibody-positive rate of over 50%, while the pig farms from where more than 100 serum samples were collected showed a *K. pneumoniae* antibody-positive rate of 35%, approximately. The positive rates of *K. pneumoniae* antibody in serum samples from pig farms of Inner Mongolia ranged from 0 to 32.23%, which were mainly concentrated in Chifeng (32.23%), Hohhot (31.58%), and Ordos (18.46%), while the positive rates of *K. pneumoniae* antibody in other areas were lower than 2% (Figure 6A; Supplementary Table S3).

### 3.8 Detection of the prevalence of *Klebsiella pneumoniae* in pigs by nested PCR

The 920 serum samples from pig farms in Jinan, Shandong and different cities of Inner Mongolia were also investigated using established nested PCR. In general, the results showed that among these samples, clear bands with expected size were observed in 176 *K. pneumoniae* serum samples, but not in other 744 samples (Supplementary Figure S1). The positive rate was 19.13% (176/920) (Figure 6B). Specifically, the positive rates in Jinan and Inner Mongolia were 35.48% (154/434) and 4.53% (22/486), respectively (Supplementary Tables S4, S5). Moreover, the positive rates in different areas of Shandong and Inner Mongolia were also different. In pig farms of Jinan, Shandong, the positive rates ranged from 13.33 to 73.33% (Supplementary Table S4). In pig farms of Inner Mongolia, the positive rates ranged from 0 to 7.02% (Figure 6A; Supplementary Table S5).

### 3.9 Determination of factors correlated to *Klebsiella pneumoniae* infection rate

**Figure 7:**
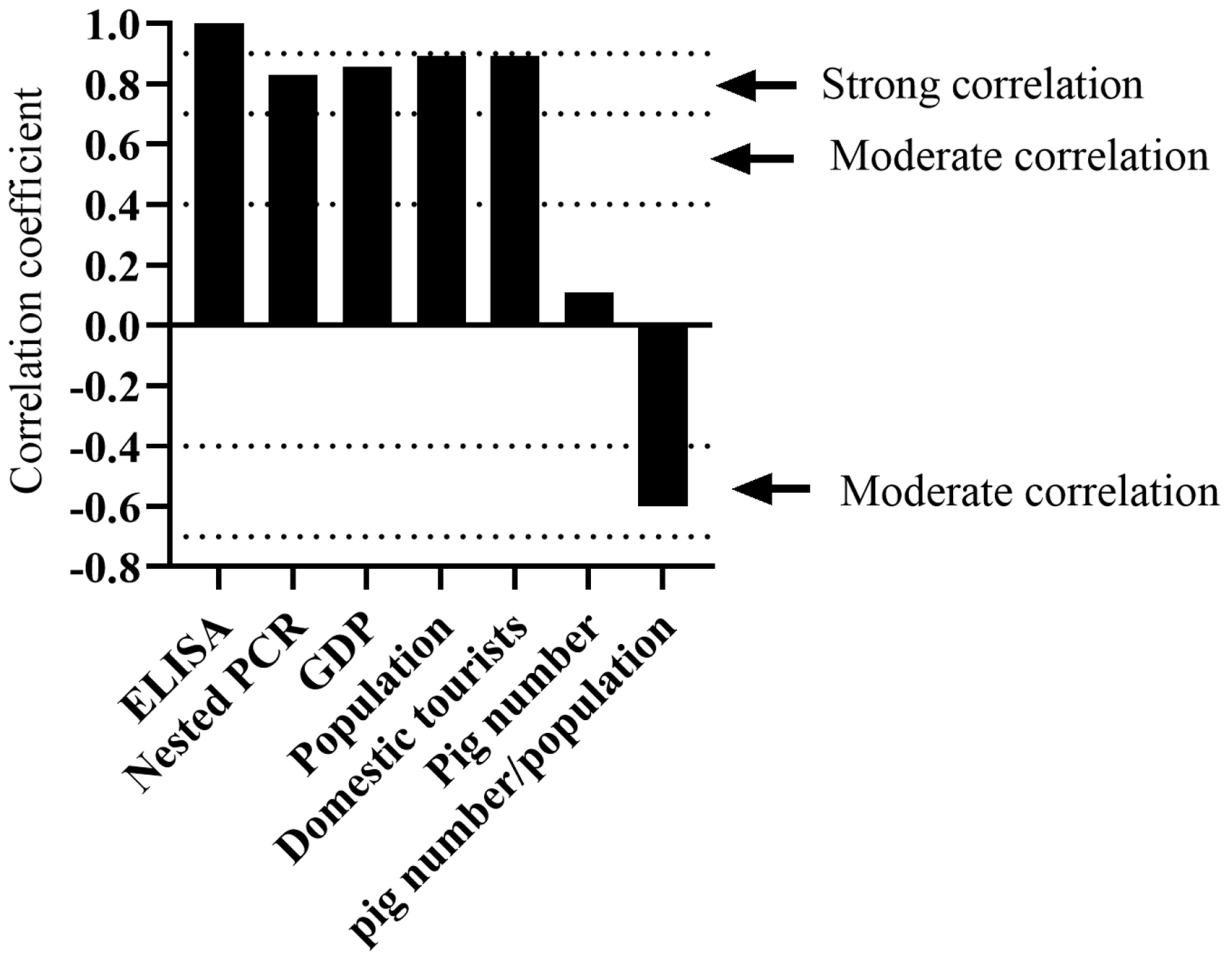
Determination of factors correlated to *K. pneumoniae* infection rate by Spearman correlation analysis. Correlation coefficient between 0.00–0.10, negligible correlation; 0.10–0.39, weak correlation; 0.40–0.69, moderate correlation; 0.70–0.89, strong correlation; 0.90–1.00, very strong correlation.

The *K. pneumoniae* infection rate in pig’s serum from Jinan, Shandong is much higher than that from other cities of Inner Mongolia (Figure 6A). Since the cities with higher positivity rates such as Jinan and Hohhot tend to be more developed, we wonder if the *K. pneumoniae* infection rate is associated with human activities including gross population, population mobility, Gross Domestic Product (GDP), and breeding density (number of pigs/population). As expected, high infection rates were mainly observed in Jinan, Chifeng, Hohhot, and Ordos, which have higher gross population, GDP, and population mobility (the number of tourists) than other cities (Table 3). Accordingly, Spearman correlation analysis demonstrated a strong positive correlation between *K. pneumoniae* infection rate and gross population, GDP, and the number of tourists. On the other hand, a moderate negative correlation between *K. pneumoniae* infection rate and breeding density was observed (Table 3; Figure 7). Overall, these results suggest that the prevalence of *K. pneumoniae* in pigs is positively associated with indicated human activities. The more frequent human activities, the higher incidence of *K. pneumoniae* infection in pigs.

**Table 3:** Correlation coefficient of ELISA and different factors by Spearman correlation analysis.

Cities	Positive rate		GDP^†^	Population^†^	Domestic tourists^†^	Pig number	Breeding density
	ELISA	Nested PCR	(0.1 billion)	(10,000 person)	(10,000 person-times)	(10,000)	(Pig number/population)
Jinan	41.24%	35.48%	10140.9	920.24	6037.9	131.95	6.97416
Chifeng	32.23%	5.79%	1763.6	403.13	1383.92	139.19	2.89626
Hohhot	31.58%	7.02%	2800.68	345.42	1820.36	41.03	8.41872
Ordos	18.46%	6.15%	3533.66	215.56	1580.84	28.29	7.61965
Tongliao	1.61%	0.00%	1276.64	286.75	1158.32	157.83	1.81683
Hulun Buir	1.55%	5.43%	1172.2	223.63	781.16	34.53	6.4764
Hinggan League	0.00%	0.00%	547.92	141.32	681.1	53.04	2.6644
Correlation coefficient	1	0.829	0.857	0.893	0.893	0.109	−0.6
*P*	0.0004	0.03	0.023	0.012	0.012	0.816	0.154

^†^GDP, population, and domestic tourists are calculated using the data in the year 2020. Data of Jinan is obtained from the Jinan statistical yearbook 2021 published by Jinan government (http://jntj.jinan.gov.cn/art/2021/10/11/art_27523_4743879.html). Data of others cities is obtained from the Statistical Bureau of Inner Mongolia (http://tj.nmg.gov.cn/datashow/easyquery/easyquery.htm?cn=B0103). GDP, Gross Domestic Product.

### 3.10 Comparison of results between the established ELISA and nested PCR

We found that the consistency between positive samples identified by ELISA and nested PCR was not high. As shown in Supplementary Table S6, there were 559 negative samples as detected by both methods, indicating that 60.76% of pigs have never been infected with *K. pneumoniae*. There were 66 positive samples as detected by both methods, indicating that 7.17% of pigs are infected with *K. pneumoniae*. Moreover, there were 110 samples showed *K. pneumoniae* antibody negative as detected by established ELISA, but identified as positive by nested PCR, which may indicate that 11.96% of pigs might be infected by *K. pneumoniae* at an early-stage. Identified as positive by established ELISA, while negative by nested PCR were observed in 185 samples, which may indicate that 20.11% of pigs have endured the infection of *K. pneumoniae*.

## 4 Discussion

*Klebsiella pneumoniae* is an important opportunistic and zoonotic pathogen that causes various diseases in humans and animals (Bowring et al., 2017; Bidewell et al., 2018). However, the pathogenicity and epidemiology of *K. pneumoniae* in pigs is still unknown due to the lack of rapid and sensitive diagnosis techniques. In this study, we show that infection by a human origin *K. pneumoniae* led to mild lung injury of pigs. Epidemic detection of *K. pneumoniae* by an established indirect ELISA and nested PCR demonstrate a high infection rate in pigs, which is associated with local population, GDP, human activities, and breeding density. These results provide valuable information for the illustration of transmission and control of diseases caused by *K. pneumoniae* both in humans and pigs.

Though *K. pneumoniae*-induced septicemia have been observed, infections by *K. pneumoniae* in pigs are usually not fatal and rarely reported in veterinary medicine (Bowring et al., 2017; Bidewell et al., 2018). The effect of *K. pneumoniae* infection on pig health have been neglected. However, we speculate that *K. pneumoniae* infection may also impair pig health, especially in lung, even though the symptoms are subclinical or even asymptomatic. As expected, we observed mild lung injury and body weight loss upon *K. pneumoniae* infection in pigs (Figure 2). Importantly, the *K. pneumoniae* strain used in infection experiment in pigs is an hypervirulent serotype K2 strain that was isolated from human. According to our knowledge, this is the first report on the virulence of *K. pneumoniae* from a human origin on lung of pigs. Moreover, *K. pneumoniae* have been isolated from lung samples together with other common pathogens that cause pneumoniae in pigs, such as *E. coli* (Lai et al., 2018) and porcine reproductive and respiratory syndrome virus (PRRSV) (Yan et al., 2020). We assume that *K. pneumoniae* may contributes to the pathogeneses of these pathogens.

According to our knowledge, there’s no commercial kits for the rapid detection of *K. pneumoniae* antibodies in pig serum. Conventional techniques including PCR are commonly utilized for detection and identification of *K. pneumoniae* (Chen et al., 2021). ELISA is a sensitive, specific, and one of the most common serological methods for the pathogen detection in veterinary medicine. Compared to conventional PCR techniques, it does not require expensive instruments, which makes it a convenient tool for rapid detection of pathogens. In this study, we have established an indirect ELISA method which displayed high sensitivity, reproducibility, and specificity in the assays. Since there’s no vaccines for pigs in China against *K. pneumoniae* infection, the established ELISA can be utilized for the diagnosis of *K. pneumoniae* infection by detecting whether the serum contain the specific antibodies. However, a major drawback of this method is that it may requires large amounts of rKHE protein as coating antigens for large-scale serum detections. Thus, a developed protein purification method might be needed. Moreover, evaluation of the prevalence of *K. pneumoniae* might be inadequate since the established ELISA detects anti-rKHE antibody which takes certain time to be produced during infection process by host. Thus, infections at an early stage might be overlooked.

To overcome these drawbacks, a nested PCR that detects nucleic acids of *khe* was developed. Compared conventional PCR, nested PCR has higher sensitivity and specificity in terms of amplifying a cDNA copy of an mRNA present at very low abundance in samples (Green and Sambrook, 2019). In line with these speculations, ELISA and nested PCR gave different results of positive rates in serum samples from both areas, suggesting the pigs were infected with *K. pneumoniae* at different infection stages (Pinedo et al., 2008). ELISA-positive results indicated that the pigs have been infected with *K. pneumoniae* for some time or at a late stage of infection, while nested PCR-positive results indicated that the animal are infected with *K. pneumoniae* at present. Analysis of the results from the both established methods may be needed to better evaluate the prevalence of *K. pneumoniae* in animals.

Most of the infections caused by *K. pneumoniae* have been observed in diverse animals including cattle, goat, horse, and cat, but not many in pigs (Brisse and Duijkeren, 2005; Ribeiro et al., 2022). In this study, a total of 920 serum samples from Shandong and Inner Mongolia of China were detected using the established methods. The positive rates of *K. pneumoniae* antibody in both areas were quite high (Figure 6B), indicating *K. pneumoniae* is a major emerging pathogen that prevalent in pigs. Our result is consistent with a previous study which showed *K. pneumoniae* was the sixth most frequently encountered pathogen in swine (Zou et al., 2011). Considering the fact that *K. pneumoniae* can be transmitted between humans and animals via food chain and occupational contact (Founou et al., 2018; Yang F. et al., 2019; Leangapichart et al., 2021), we hypothesize that the domestic animals may represent a source of the pathogenic and multidrug-resistant *K. pneumoniae* to humans or vice versa.

Moreover, we observed that the *K. pneumoniae* infection rate varied dramatically in different regions and pig farms (Supplementary Tables S2–S5). Regions with high population density and GDP had higher infection rate than that with low population density and GDP. Moreover, *K. pneumoniae* infection rate is positively correlated with gross population, GDP, and the number of tourists (Figure 7), but negatively correlated with breeding density, suggesting that the prevalence of *K. pneumoniae* in pigs is associated with human activities.

In conclusion, our study demonstrated that *K. pneumoniae* infection impaired pig health. Rapid detection of *K. pneumoniae* by the established indirect ELISA and nested PCR indicated a high infection rate in pigs, which is correlated with local population, GDP, human activities and breeding density. These results suggested the established methods can be applied for rapid clinical diagnoses to evaluate the prevalence of *K. pneumoniae* infection both in animals and humans, and highlighted the effect of *K. pneumoniae* infection on pig health to which needs to be paid more attentions in veterinary medicine.

## Data availability statement

The original contributions presented in the study are included in the article/Supplementary material, further inquiries can be directed to the corresponding authors.

## Ethics statement

The animal study was approved by the Institutional Animal Care and Use Committee of Jilin University. The study was conducted in accordance with the local legislation and institutional requirements.

## Author contributions

ZW: Writing – original draft, Writing – review & editing. NL: Writing – original draft, Writing – review & editing. ZL: Writing – original draft. JW: Writing – original draft. ML: Writing – original draft. MQ: Writing – original draft. SW: Writing – review & editing. TW: Methodology, Writing – original draft. YG: Resources, Writing – original draft. JZ: Methodology, Writing – original draft. HJ: Methodology, Writing – original draft. RX: Resources, Writing – original draft. CS: Resources, Writing – original draft. XF: Writing – original draft. JG: Methodology, Supervision, Writing – review & editing. WH: Writing – review & editing. FL: Writing – original draft, Writing – review & editing. LL: Writing – original draft, Writing – review & editing.

## Footnotes

^1^ www.snapgene.com

## References

[ref1] Barbier E. Rodrigues C. Depret G. Passet V. Gal L. Piveteau P.. (2020). The ZKIR assay, a real-time PCR method for the detection of *Klebsiella pneumoniae* and closely related species in environmental samples. Appl. Environ. Microbiol. 86:2711. doi: 10.1128/aem.02711-19, PMID: 32005732 PMC7082575

[ref2] Bidewell C. A. Williamson S. M. Rogers J. Tang Y. Ellis R. J. Petrovska L.. (2018). Emergence of *Klebsiella pneumoniae* subspecies pneumoniae as a cause of septicaemia in pigs in England. PLoS One 13:e0191958. doi: 10.1371/journal.pone.0191958, PMID: 29470491 PMC5823397

[ref3] Bowring B. G. Fahy V. A. Morris A. Collins A. M. (2017). An unusual culprit: *Klebsiella pneumoniae* causing septicaemia outbreaks in neonatal pigs? Vet. Microbiol. 203, 267–270. doi: 10.1016/j.vetmic.2017.03.018, PMID: 28619154

[ref4] Brisse S. Duijkeren E. (2005). Identification and antimicrobial susceptibility of 100 Klebsiella animal clinical isolates. Vet. Microbiol. 105, 307–312. doi: 10.1016/j.vetmic.2004.11.010, PMID: 15708829

[ref5] Cai R. Wu M. Zhang H. Zhang Y. Cheng M. Guo Z.. (2018). A smooth-type, phage-resistant *Klebsiella pneumoniae* mutant strain reveals that OmpC is indispensable for infection by phage GH-K3. Appl. Environ. Microbiol. 84:1585. doi: 10.1128/aem.01585-18, PMID: 30171001 PMC6193389

[ref6] Chen Z. Liu M. Cui Y. Wang L. Zhang Y. Qiu J.. (2014). A novel PCR-based genotyping scheme for clinical *Klebsiella pneumoniae*. Future Microbiol. 9, 21–32. doi: 10.2217/fmb.13.137, PMID: 24328378

[ref7] Chen R. Shang H. Niu X. Huang J. Miao Y. Sha Z.. (2021). Establishment and evaluation of an indirect ELISA for detection of antibodies to goat *Klebsiella pneumonia*. BMC Vet. Res. 17:107. doi: 10.1186/s12917-021-02820-1, PMID: 33663505 PMC7934495

[ref8] Dong D. Liu W. Li H. Wang Y. Li X. Zou D.. (2015). Survey and rapid detection of *Klebsiella pneumoniae* in clinical samples targeting the rcsA gene in Beijing, China. Front. Microbiol. 6:519. doi: 10.3389/fmicb.2015.00519, PMID: 26052327 PMC4440914

[ref9] Escobar Pérez J. A. Olarte Escobar N. M. Castro-Cardozo B. Valderrama Márquez I. A. Garzón Aguilar M. I. Martinez de la Barrera L.. (2013). Outbreak of NDM-1-producing *Klebsiella pneumoniae* in a neonatal unit in Colombia. Antimicrob. Agents Chemother. 57, 1957–1960. doi: 10.1128/aac.01447-12, PMID: 23357776 PMC3623329

[ref10] Founou L. L. Founou R. C. Allam M. Ismail A. Djoko C. F. Essack S. Y. (2018). Genome sequencing of extended-Spectrum β-lactamase (ESBL)-producing *Klebsiella pneumoniae* isolated from pigs and abattoir Workers in Cameroon. Front. Microbiol. 9:188. doi: 10.3389/fmicb.2018.00188, PMID: 29479347 PMC5811526

[ref11] Green M. R. Sambrook J. (2019). Nested polymerase chain reaction (PCR). Cold Spring Harb. Protoc. 2019:pdb.prot095182. doi: 10.1101/pdb.prot095182 30710024

[ref12] He Y. Guo X. Xiang S. Li J. Li X. Xiang H.. (2016). Comparative analyses of phenotypic methods and 16S rRNA, khe, rpoB genes sequencing for identification of clinical isolates of *Klebsiella pneumoniae*. Antonie Van Leeuwenhoek 109, 1029–1040. doi: 10.1007/s10482-016-0702-9, PMID: 27147066

[ref13] Huang Y. Li J. Wang Q. Tang K. Li C. (2022). Rapid detection of KPC-producing *Klebsiella pneumoniae* in China based on MALDI-TOF MS. J. Microbiol. Methods 192:106385. doi: 10.1016/j.mimet.2021.106385, PMID: 34843862

[ref14] Jeong E. S. Lee K. S. Heo S. H. Seo J. H. Choi Y. K. (2013). Rapid identification of *Klebsiella pneumoniae*, *Corynebacterium kutscheri*, and *Streptococcus pneumoniae* using triplex polymerase chain reaction in rodents. Exp. Anim. 62, 35–40. doi: 10.1538/expanim.62.35 23357944

[ref15] Jian-Li W. Yuan-Yuan S. Shou-Yu G. Fei-Fei D. Jia-Yu Y. Xue-Hua W.. (2017). Serotype and virulence genes of *Klebsiella pneumoniae* isolated from mink and its pathogenesis in mice and mink. Sci. Rep. 7:17291. doi: 10.1038/s41598-017-17681-8, PMID: 29230010 PMC5725566

[ref16] Jin Z. Wang Z. Gong L. Yi L. Liu N. Luo L.. (2022). Molecular epidemiological characteristics of carbapenem-resistant *Klebsiella pneumoniae* among children in China. AMB Express 12:89. doi: 10.1186/s13568-022-01437-3, PMID: 35829853 PMC9279541

[ref17] Kim H. Jang J. H. Jung I. Y. Cho J. H. (2022). A novel peptide as a specific and selective probe for *Klebsiella pneumoniae* detection. Biosensors (Basel) 12:153. doi: 10.3390/bios12030153, PMID: 35323423 PMC8946155

[ref18] Ko J. H. Baek J. Y. Peck K. R. Cho S. Y. Ha Y. E. Kim S. H.. (2017). Discrepant susceptibility to gentamicin despite amikacin resistance in *Klebsiella pneumoniae* by VITEK 2 represents false susceptibility associated with the armA 16S rRNA methylase gene. J. Med. Microbiol. 66, 1448–1450. doi: 10.1099/jmm.0.000583, PMID: 28893358

[ref19] Lai C. C. Lin Y. T. Lin Y. T. Lu M. C. Shi Z. Y. Chen Y. S.. (2018). Clinical characteristics of patients with bacteraemia due to the emergence of mcr-1-harbouring Enterobacteriaceae in humans and pigs in Taiwan. Int. J. Antimicrob. Agents 52, 651–657. doi: 10.1016/j.ijantimicag.2018.08.015, PMID: 30145246

[ref20] Lalruatdiki A. Dutta T. K. Roychoudhury P. Subudhi P. K. (2018). Extended-spectrum β-lactamases producing multidrug resistance *Escherichia coli*, Salmonella and *Klebsiella pneumoniae* in pig population of Assam and Meghalaya, India. Vet. World 11, 868–873. doi: 10.14202/vetworld.2018.868-873, PMID: 30034183 PMC6048086

[ref21] Leangapichart T. Lunha K. Jiwakanon J. Angkititrakul S. Järhult J. D. Magnusson U.. (2021). Characterization of *Klebsiella pneumoniae* complex isolates from pigs and humans in farms in Thailand: population genomic structure, antibiotic resistance and virulence genes. J. Antimicrob. Chemother. 76, 2012–2016. doi: 10.1093/jac/dkab118, PMID: 33829268 PMC8283727

[ref22] Li P. Zhang D. Li H. Pang J. Guo H. Qiu J. (2020). Establishment and application of multiplex PCR for simultaneously detecting *Escherichia coli*, Salmonella, Klebsiella pneumoniae, and *Staphylococcus aureus* in minks. Front. Vet. Sci. 7:588173. doi: 10.3389/fvets.2020.588173, PMID: 33313077 PMC7704438

[ref23] Lim H. J. Kang E. R. Park M. Y. Kim B. K. Kim M. J. Jung S.. (2021). Development of a multiplex real-time PCR assay for the simultaneous detection of four bacterial pathogens causing pneumonia. PLoS One 16:e0253402. doi: 10.1371/journal.pone.0253402, PMID: 34138947 PMC8211157

[ref24] Liu Y. Liu C. Zheng W. Zhang X. Yu J. Gao Q.. (2008). PCR detection of *Klebsiella pneumoniae* in infant formula based on 16S-23S internal transcribed spacer. Int. J. Food Microbiol. 125, 230–235. doi: 10.1016/j.ijfoodmicro.2008.03.005, PMID: 18579248

[ref25] Liu D. Yang Y. Gu J. Tuo H. Li P. Xie X.. (2019). The Yersinia high-pathogenicity island (HPI) carried by a new integrative and conjugative element (ICE) in a multidrug-resistant and hypervirulent *Klebsiella pneumoniae* strain SCsl1. Vet. Microbiol. 239:108481. doi: 10.1016/j.vetmic.2019.108481, PMID: 31767086

[ref26] Mahrous S. H. El-Balkemy F. A. Abo-Zeid N. Z. El-Mekkawy M. F. El Damaty H. M. Elsohaby I. (2023). Antibacterial and anti-biofilm activities of cinnamon oil against multidrug-resistant *Klebsiella pneumoniae* isolated from pneumonic sheep and goats. Pathogens 12:1138. doi: 10.3390/pathogens12091138, PMID: 37764946 PMC10536549

[ref27] Mayoral C. Norona M. Baroni M. R. Giani R. Zalazar F. (2005). Evaluation of a nested-PCR assay for *Streptococcus pneumoniae* detection in pediatric patients with community-acquired pneumonia. Rev. Argent. Microbiol. 37, 184–188. PMID: 16502637

[ref28] Mobasseri G. Teh C. S. J. Ooi P. T. Tan S. C. Thong K. L. (2019). Molecular characterization of multidrug-resistant and extended-Spectrum Beta-lactamase-producing *Klebsiella pneumoniae* isolated from swine farms in Malaysia. Microb. Drug Resist. 25, 1087–1098. doi: 10.1089/mdr.2018.0184, PMID: 30844323

[ref29] Paczosa M. K. Mecsas J. (2016). *Klebsiella pneumoniae*: going on the offense with a strong defense. Microbiol. Mol. Biol. Rev. 80, 629–661. doi: 10.1128/mmbr.00078-15, PMID: 27307579 PMC4981674

[ref30] Pinedo P. J. Rae D. O. Williams J. E. Donovan G. A. Melendez P. Buergelt C. D. (2008). Association among results of serum ELISA, faecal culture and nested PCR on milk, blood and faeces for the detection of paratuberculosis in dairy cows. Transbound. Emerg. Dis. 55, 125–133. doi: 10.1111/j.1865-1682.2007.01009.x, PMID: 18397500

[ref31] Poirier A. C. Kuang D. Siedler B. S. Borah K. Mehat J. W. Liu J.. (2021). Development of loop-mediated isothermal amplification rapid diagnostic assays for the detection of Klebsiella pneumoniae and Carbapenemase genes in clinical samples. Front. Mol. Biosci. 8:794961. doi: 10.3389/fmolb.2021.794961, PMID: 35223985 PMC8864245

[ref32] Ribeiro M. G. de Morais A. B. C. Alves A. C. Bolanos C. A. D. de Paula C. L. Portilho F. V. R.. (2022). Klebsiella-induced infections in domestic species: a case-series study in 697 animals (1997-2019). Braz. J. Microbiol. 53, 455–464. doi: 10.1007/s42770-021-00667-0 35018603 PMC8882559

[ref33] Turton J. F. Perry C. Elgohari S. Hampton C. V. (2010). PCR characterization and typing of *Klebsiella pneumoniae* using capsular type-specific, variable number tandem repeat and virulence gene targets. J. Med. Microbiol. 59, 541–547. doi: 10.1099/jmm.0.015198-0 20110386

[ref34] Wang G. Zhao G. Chao X. Xie L. Wang H. (2020). The characteristic of virulence, biofilm and antibiotic resistance of *Klebsiella pneumoniae*. Int. J. Environ. Res. Public Health 17:6278. doi: 10.3390/ijerph17176278, PMID: 32872324 PMC7503635

[ref35] Yan X. Zeng J. Li X. Zhang Z. Din A. U. Zhao K.. (2020). High incidence and characteristic of PRRSV and resistant bacterial co-infection in pig farms. Microb. Pathog. 149:104536. doi: 10.1016/j.micpath.2020.104536, PMID: 32980472

[ref36] Yang F. Deng B. Liao W. Wang P. Chen P. Wei J. (2019). High rate of multiresistant *Klebsiella pneumoniae* from human and animal origin. Infect. Drug Resist. 12, 2729–2737. doi: 10.2147/IDR.S219155, PMID: 31564923 PMC6731983

[ref37] Yang Y. Higgins C. H. Rehman I. Galvao K. N. Brito I. L. Bicalho M. L.. (2019). Genomic diversity, virulence, and antimicrobial resistance of *Klebsiella pneumoniae* strains from cows and humans. Appl. Environ. Microbiol. 85:2654. doi: 10.1128/aem.02654-18, PMID: 30610074 PMC6414388

[ref9001] Yin-Ching C. Jer-Horng S. Ching-Nan L. Ming-Chung C. (2002). Cloning of a gene encoding a unique haemolysin from Klebsiella pneumoniae and its potential use as a species-specific gene probe. Microb Pathog 33, 1–6. doi: 10.1006/mpat.2002.0499 12127794

[ref38] Zhao W. Li S. Schwarz S. Li A. Yao H. Du X. D. (2021). Detection of a NDM-5-producing *Klebsiella pneumoniae* sequence type 340 (CG258) high-risk clone in swine. Vet. Microbiol. 262:109218. doi: 10.1016/j.vetmic.2021.109218, PMID: 34481222

[ref39] Zou L. K. Wang H. N. Zeng B. Zhang A. Y. Li J. N. Li X. T.. (2011). Phenotypic and genotypic characterization of β-lactam resistance in *Klebsiella pneumoniae* isolated from swine. Vet. Microbiol. 149, 139–146. doi: 10.1016/j.vetmic.2010.09.030, PMID: 21035968

