# Supplementary material for: Development and application of an indirect ELISA and nested PCR for the epidemiological analysis of Klebsiella pneumoniae among pigs in China
Source: Front Microbiol. 2024 Jan 8;14:1329609. doi: 10.3389/fmicb.2023.1329609 (PMC10803024; doi:10.3389/fmicb.2023.1329609)
Supplement: Supplementary file 1 [file Data_Sheet_1.docx]

Supplementary Material

# Supplementary Figures and Tables

## Supplementary Tables

**Table S1 Primers, plasmids and bacteria used in this study**

| Primers | Description and source |
| --- | --- |
| KHE (F) | CCAAGCTTATATGAAACGACCTGATTGCATTC |
| KHE (R) | CCCTCGAGCTTTTTCCGCGGCTTACCGTC |
| KHE 1 (F) | CCAAGCTTATATGAAACGACCTGATTGCATTC |
| KHE 1 (R) | CCCTCGAGCTTTTTCCGCGGCTTACCGTC |
| KHE 2 (F) | AGAGCGATGAGGAAGAGTTCA |
| KHE 2 (R) | CGGGTAATAAATGCGGTTGTA |
| Plasmids | Description |
| pET28a | Stored in our laboratory |
| Strains | Description |
| DH5α | Sangon Biotech |
| BL21(DE3) | Sangon Biotech |
| *K. pneumoniae* strain KP-Q1 | Resistance: Ampicillin; serotype: K2 |
| *K. pneumoniae* strain K36 | Serotype: K2; hypervirulent *K. pneumoniae* (hvKP) (Cai et al., 2018) |

The enzyme digestion site was indicated as underlined.

**Table S2 K. pneumoniae antibody-positive rate in pig farms in Shandong by established ELISA**

| Farm number | The number of positive samples | Total number of samples | Positive rate (%) |
| --- | --- | --- | --- |
| 1 | 5 | 15 | 33.33% |
| 2 | 0 | 15 | 0.00% |
| 3 | 12 | 15 | 80.00% |
| 4 | 15 | 15 | 100.00% |
| 5 | 5 | 40 | 12.50% |
| 6 | 40 | 114 | 35.09% |
| 7 | 12 | 15 | 80.00% |
| 8 | 14 | 15 | 93.33% |
| 9 | 13 | 15 | 86.67% |
| 10 | 63 | 175 | 36% |
| total | 179 | 434 | 41.24% |

**Table S3 K. pneumoniae antibody-positive rate in pig farms in Inner Mongolia by established ELISA**

| Cities | The number of positive samples | Total number of samples | Positive rate (%) |
| --- | --- | --- | --- |
| Hulun Buir | 2 | 129 | 1.55% |
| Hinggan League | 0 | 52 | 0.00% |
| Chifeng | 39 | 121 | 32.23% |
| Tongliao | 1 | 62 | 1.61% |
| Ordos | 12 | 65 | 18.46% |
| Hohhot | 18 | 57 | 31.58% |
| total | 72 | 486 | 14.81% |

**Table S4 K. pneumoniae positive rate in pig farms in Shandong by nested PCR**

| Farm number | The number of positive samples | Total number of samples | Positive rate (%) |
| --- | --- | --- | --- |
| 1 | 5 | 15 | 33.33% |
| 2 | 2 | 15 | 13.33% |
| 3 | 8 | 15 | 53.33% |
| 4 | 3 | 15 | 20.00% |
| 5 | 23 | 40 | 57.50% |
| 6 | 52 | 114 | 45.61% |
| 7 | 11 | 15 | 73.33% |
| 8 | 7 | 15 | 46.67% |
| 9 | 2 | 15 | 13.33% |
| 10 | 41 | 175 | 23.43% |
| Total | 154 | 434 | 35.48% |

**Table S5 K. pneumoniae positive rate in pig farms in Inner Mongolia by nested PCR**

| Cities | The number of positive samples | Total number of samples | Positive rate (%) |
| --- | --- | --- | --- |
| Hulun Buir | 7 | 129 | 5.42% |
| Hinggan League | 0 | 52 | 0.00% |
| Chifeng | 7 | 121 | 5.78% |
| Tongliao | 0 | 62 | 0.00% |
| Ordos | 4 | 65 | 6.15% |
| Hohhot | 4 | 57 | 7.02% |
| total | 22 | 486 | 4.53% |

**Table S6 Comparison of ELISA and Nest PCR results**

|  | ELISA positive | ELISA negative | total |
| --- | --- | --- | --- |
| nested PCR positive | 66 | 110 | 176 |
| nested PCR negative | 185 | 559 | 744 |
| total | 251 | 669 | 920 |

## Supplementary Figures


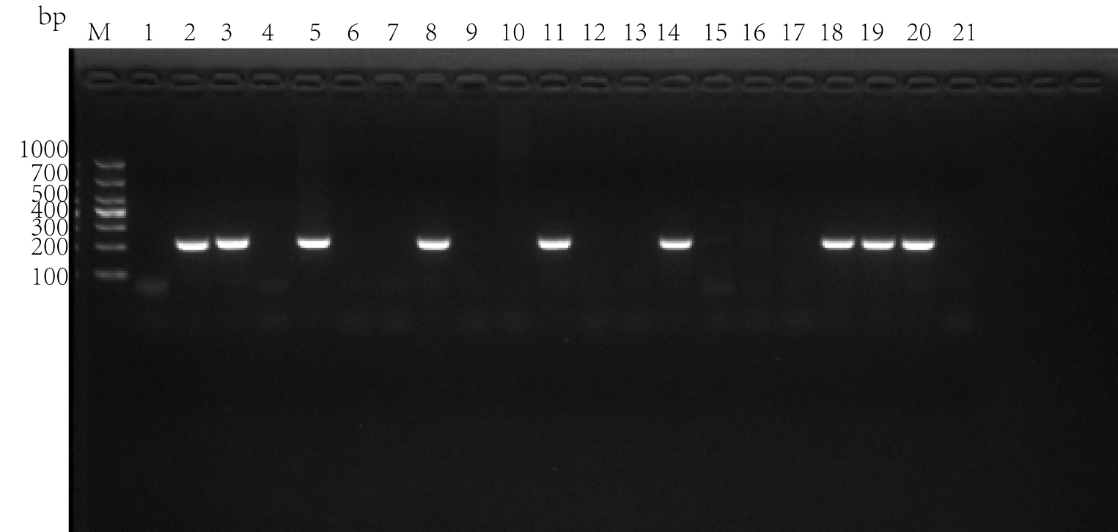


**Supplementary Figure 1.** Detection of K. pneumoniae khe in selected 21 serum samples by nested PCR. M, DNA Marker; 1, negative serum control; 2-21, clinical serum samples. Expected size: 210 bp.
